# Exposure of Larval Zebrafish to the Insecticide Propoxur Induced Developmental Delays that Correlate with Behavioral Abnormalities and Altered Expression of *hspb9* and *hspb11*

**DOI:** 10.3390/toxics7040050

**Published:** 2019-09-21

**Authors:** Jeremiah N. Shields, Eric C. Hales, Lillian E. Ranspach, Xixia Luo, Steven Orr, Donna Runft, Alan Dombkowski, Melody N. Neely, Larry H. Matherly, Jeffrey W. Taub, Tracie R. Baker, Ryan Thummel

**Affiliations:** 1 Institute of Environmental Health Sciences, Center for Urban Responses to Environmental Stressors, Wayne State University, Detroit, MI 48201, USA; jshields@wayne.edu (J.N.S.); tracie.baker@wayne.edu (T.R.B.); 2 Barbara Ann Karmanos Cancer Institute, Detroit, MI, 48201, USA; ehales1993@gmail.com (E.C.H.); sorr02@yahoo.com (S.O.); 3 Department of Ophthalmology, Visual and Anatomical Sciences, Wayne State University School of Medicine, Detroit, MI 48201, USA; lillian.ranspach@wayne.edu (L.E.R.); xluo@med.wayne.edu (X.L.); 4 Department of Biochemistry, Microbiology and Immunology, Wayne State University School of Medicine, Detroit, Michigan 48201, USA; ak1541@wayne.edu (D.R.); melody.neely@maine.edu (M.N.N.); 5 Department of Pediatrics, Wayne State University School of Medicine, Detroit, MI 48201, USA; domski@wayne.edu (A.D.);; 6 Barbara Ann Karmanos Cancer Institute, Department of Oncology, Wayne State University School of Medicine, Detroit, MI 48201, USA; matherly@karmanos.org; 7 Department of Pharmacology, Wayne State University, Detroit, MI 48201, USA

**Keywords:** zebrafish, pesticide, insecticide, propoxur, Baygon™, developmental delays, *hspb9*, *hspb11*, behavior, light-dark test

## Abstract

Recent studies suggest that organophosphates and carbamates affect human fetal development, resulting in neurological and growth impairment. However, these studies are conflicting and the extent of adverse effects due to pesticide exposure warrants further investigation. In the present study, we examined the impact of the carbamate insecticide propoxur on zebrafish development. We found that propoxur exposure delays embryonic development, resulting in three distinct developmental stages: no delay, mild delay, or severe delay. Interestingly, the delayed embryos all physically recovered 5 days after exposure, but behavioral analysis revealed persistent cognitive deficits at later stages. Microarray analysis identified 59 genes significantly changed by propoxur treatment, and Ingenuity Pathway Analysis revealed that these genes are involved in cancer, organismal abnormalities, neurological disease, and hematological system development. We further examined *hspb9* and *hspb11* due to their potential roles in zebrafish development and found that propoxur increases expression of these small heat shock proteins in all of the exposed animals. However, we discovered that less significant increases were associated with the more severely delayed phenotype. This raises the possibility that a decreased ability to upregulate these small heat shock proteins in response to propoxur exposure may cause embryos to be more severely delayed.

## 1. Introduction

According to recent reports by the United States Environmental Protection Agency (US EPA), over 1.1 billion pounds of conventional pesticides were used in the United States in 2012 [1]. Worldwide totals are near 6 billion pounds of usage and continues to grow annually [1]. Chlorpyrifos, acephate, and malathion account for the most widely used organophosphate (OP) insecticides in the U.S., contributing to millions of pounds of pesticides released into the environment [1]. Due to environmental toxicity concerns, usage of OP insecticides has shifted to newer alternatives such as carbamates, including carbaryl (Sevin™) and propoxur (Baygon™) [1,2]. Carbaryl is more widely used both in agriculture and in the home while propoxur is restricted to residential pest control [1,3,4].

Research suggests that fetal development is the window of susceptibility for adverse effects of pesticide exposures, although work in this area is conflicting. In particular, the fetal brain is sensitive to toxicants due to rapid growth and development [5,6,7]. Previous studies have shown that OP and carbamate insecticides are neurotoxic due to inhibition of acetylcholinesterase (AChE), resulting in increased levels of the neurotransmitter acetylcholine in the synaptic cleft, impeding cholinergic neurotransmission [8]. OP exposure resulted in abnormal reflexes in newborn infants [9] as well as delays in cognitive development with impairment in both mental and psychomotor development by 36 months of age [10]. However, exposure of Sprague–Dawley rats to the OP diazinon significantly inhibited maternal AChE activity with minimal effects on fetal AChE levels [11]. This suggests that neurotoxic effects of OP insecticides might be independent of AChE inhibition in fetal brain or might be related to the type or stability of the pesticide. Interestingly, significant decreases in both birth weight and length of human infants prenatally exposed to either chlorpyrifos or the combination of chlorpyrifos and diazinon has also been reported [12]. However, other studies found no significant correlation between prenatal OP insecticide exposure and fetal growth [13]. Thus, although there is some evidence that OP insecticide exposure negatively affects fetal growth and neurological impairment, the nature of the conflicting studies warrants further investigation. 

Carbamate insecticides have largely replaced OP insecticides for residential use [1]. Propoxur is used worldwide as a residential pest control for cockroaches, flies, mosquitoes, and lawn and turf insects; in control of the spread of malaria; and in flea collars for pets [4]. Ostrea Jr. et al. found that the most common prenatal exposure of infants born to mothers in the Philippines was exposure to propoxur. Propoxur levels were greater than other insecticides in cord plasma and meconium and reached concentrations of 0.33 to 0.77 μg/mL, respectively [14]. A follow-up study found that prenatal propoxur exposure correlated with significantly impaired motor development by 2 years of age [15]. Similar to chlorpyrifos, propoxur had similar albeit less significant effects on birth length in the study by Whyatt et al. [12]. These effects were largely replicated in a rodent model of propoxur exposure, wherein rat dams orally exposed to propoxur exhibited decreased number of pups and birth weights [16,17]. 

Zebrafish (*Danio rerio*) is an excellent model to examine the effects of pesticide exposure on development due to external embryo development, large clutch sizes, and transparency of the embryo [18]. Teratogenicity of carbamate exposure in zebrafish embryos is well documented. Work by Todd et al. found that carbaryl (Sevin™) had minimal effects on embryo size but dose-dependently delayed development by 24–72 h and normal hatching time from 72 hpf to 96–144 hpf [19]. The concentrations of carbaryl used in this study were not indicated, possibly due to poor solubility in water (0.01%). Detailed work by Lin et al. found minimal carbaryl embryo lethality at 10 μg/mL but observed accumulation of red blood cells in the head and pericardial and yolk sac edema [20]. Carbaryl neither had an effect on cardiac looping of the heart tube nor affected ventricular or atrium morphology but significantly depressed heart rate [20]. Schock et al. reported similar cardiac effects [21]. Carbaryl-treated embryos had severe tail curvature and complete paralysis by 48 hpf [21]. Reduced numbers of gefilitin 1 positive spinal cord neurons was observed, and motor axon pathfinding was impaired [21]. Delayed development was also noted [21]. Embryo-wide carbaryl-induced cell death was observed; however, the 20 μg/mL dose of carbaryl used in this study exhibited an overall high mortality [21].

In addition to the physical abnormalities induced by insecticides, there also exists several notable gene expression alterations. The heat shock proteins are an important family of proteins that are produced by cells in response to stressful events or exposures. Many members of this family (Hsp60, Hsp70, and Hsp90) perform chaperone functions to stabilize newly folded proteins or refold proteins damaged by cell stress. Scheil et al. found Hsp70 levels to have a sensitive window during development and was subjected to upregulation in response to chlorpyrifos [22]. Liu and others found Hsp60 was upregulated after chlorpyrifos exposure, suggesting an adaptive protein protective role in response to environmental toxicants [23]. In another chlorpyrifos study, Garcia-Reyero et al. found differential levels of *hsp60*, *hsp70*, and *hsp90* based on a model of mild, moderate, and severe chlorpyrifos poisoning [24]. The results from this study showed the temporal relationship between the toxicant-exposed phenotype and the resulting upregulation of the heat shock genes. Together, these studies highlight progress made in understanding the cellular mechanisms behind insecticide toxicity, but further studies will still be required. 

Due to the shift toward carbamate usage in the home and worldwide use of propoxur, we decided to further investigate the impact of this prototypical carbamate insecticide on zebrafish development. We found that propoxur exposure significantly delayed embryonic development for a brief and defined window in a subset of zebrafish exposed to the insecticide. Interestingly, delayed animals recovered post-hatching to a general physical appearance of untreated control animals. Behavior analysis of “recovered” animals revealed abnormal neurological responses. Microarray and pathway analysis revealed transcriptomic changes involved in cancer, organismal abnormalities, neurological disease, and hematological system development. This analysis also identified *hspb9* and *hspb11* as genes that were upregulated by propoxur treatment. Expression of both *hspb9* and *hspb11* inversely correlated with the severity of propoxur-induced developmental delay, suggesting that an upregulation of these proteins protects the developing embryo from environmental stresses.

## 2. Materials and Methods 

### 2.1. Fish Maintenance

The wild-type *AB* strain of zebrafish were used for these studies [25]. All procedures were approved by The Institutional Animal Care and Use Committee at Wayne State University (Protocol #IACUC-18-03-0589; Date of approval: 14 May 2019). 

### 2.2. Propoxur Treatment

**Figure 1:**
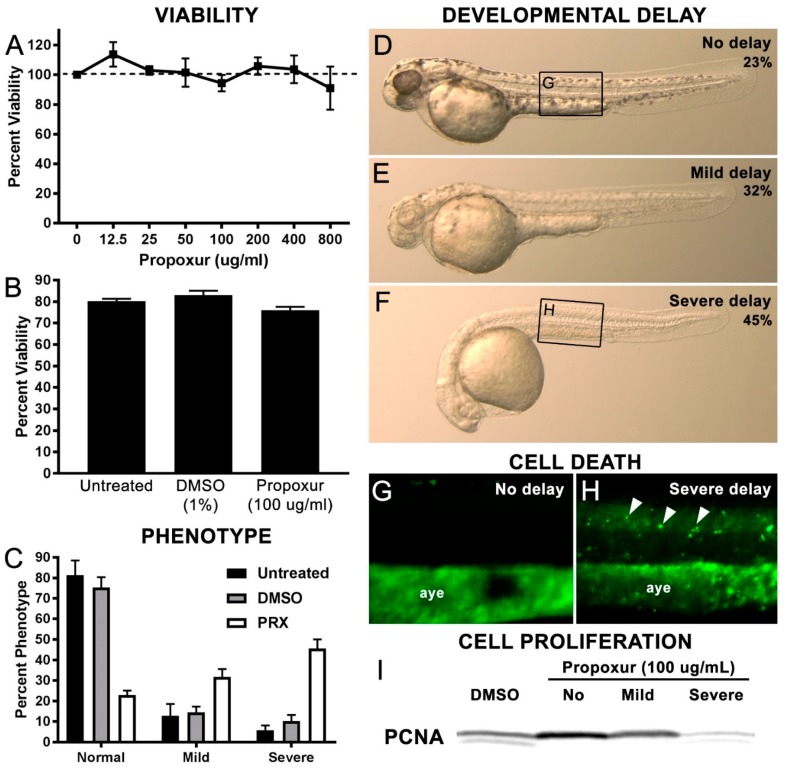
Propoxur exposure during zebrafish development results in three distinct developmental delay phenotypes: (**A**) Percent viability of treated embryos at varying propoxur concentrations; (**B**) percent viability of embryos in untreated, 1% DMSO-treated, and 100 ug/mL propoxur-treated groups; (**C**) percent of delay phenotypes present in untreated, DMSO-treated, or propoxur-treated groups; visual representation of phenotypic groups at 40 hpf: (**D**) no delay, (**E**) mild delay, and (**F**) severe delay. (**G**,**H**) Acridine orange uptake shows an increase in fluorescent puncta (arrowheads) associated with the severe delay group compared with the no delay group. These puncta are distinct from the autofluorescence observed in the anal yolk extension (aye) in both groups. The panel insets for Figure 1G,H are shown in Figure 1D,F, respectively; (**I**) Western blot analysis of the G1/S protein Proliferating Cell Nuclear Antigen (PCNA) shows reduced PCNA expression in the severe delay groups, suggestive of reduced cell proliferation.

As an initial screening of propoxur toxicity, zebrafish embryos were manually dechorionated at developmental stages between 15–16 h postfertilization and were immediately treated with either 0.125% DMSO (control group) or propoxur (experimental group) at 12.5, 25, 50, 100, 200, 400, and 800 μg/mL. At 40 hpf, dead embryos were removed and quantified (Figure 1A) and the viable embryos were washed three times with fresh embryo medium to remove all traces of propoxur and DMSO. The control and experimental fish were then screened by a scientist who did not perform the exposures and who was blinded to the treatment groups. The fish were separated into three phenotypic groups based on the degree of developmental delay using standard zebrafish staging: no delay (40 hpf), mild delay (30–36 hpf), and severe delay (16–26 hpf). Based on the relatively uniform distribution of each phenotype observed within the 100 μg/mL exposure group (Supplemental Figure S1), these experiments were repeated at this dose for subsequent experimental analysis.

### 2.3. Behavior Analysis Procedure

Behavior analysis was performed on fish at 7 days postfertilization (dpf). On the day prior, fish were transferred to individual wells of a 24-well plate (1.65-cm diameter wells) for acclimation (Supplemental Figure S2). Behavior analysis was conducted between 13:00 and 16:00 the following day using a semi-high-throughput behavioral tracking platform [26]. The 24-well plate containing the fish was transferred to the DanioVision Observation Chamber (Noldus Information Technology, Wageningen, Netherlands) (Supplemental Figure S2), and a steady flow of water was supplied to the chamber via the DanioVision Temperature Control Unit to keep the fish at a constant 28.5 ± 0.5 °C during the procedure. The fish were tracked live by a Basler Gen1 Camera (Basler acA1300-60) using the EthoVision XT 13 software (Supplemental Figure S2). The resolution was set at 1280 × 960, and the frame rate was set at 25.

Parameters for the experiment were as follows: 12-min acclimation period inside the DanioVision Observation Chamber in the dark, followed by an alternating cycle of 3-min intervals of light and dark stimuli repeated a total of 4 times. Analysis was done using EthoVision XT 13 software. Heatmaps were generated every 3 min to track the overall movement of the fish during the different intervals. Data points were exported to Excel to allow for analysis and generation of graphs. The comparison of distance traveled in light versus dark was investigated. The average velocity of the fish was also considered. Finally, in order to understand the ability of the fish to recover from the alternating stimuli, rebound line graphs and bar graphs investigating the distance traveled during the first light and dark interval versus the last light and dark interval were produced. The *n* value for each group was as follows: DMSO control = 40, no delay = 39, mild delay = 42, and severe delay = 41. Statistical differences between the groups were performed by a one-way ANOVA followed by a Bonferroni post hoc test (*p* < 0.05). 

### 2.4. Acridine Orange (AO) Staining 

Embryos were treated with or without 0.2 mM phenylthiourea (PTU) (Sigma-Aldrich, St. Louis, MO, USA) prior to propoxur treatment. Afterwards, live embryos were stained with 10 μg/mL of acridine orange (AO) [27] (Sigma-Aldrich, St. Louis, MO, USA) in E3 medium and then incubated for 30 min at 30 °C. Embryos were washed three times with fresh E3 medium and then anesthetized in 2-phenoxyethanol prior to microscopy. 

### 2.5. Microscopy

Live embryos were anesthetized in 2-phenoxyethanol prior to microscopy. Images were captured on a Spot digital camera (Diagnostic Instruments; Sterling Heights, MI, USA) attached to a Leica M165 FC stereomicroscope.

### 2.6. Heat Shock Treatment

To confirm that the expressions of *hspb9* and *hspb11* were upregulated in response to heat shock, pools of 24 hpf embryos (~50 per treatment) were either left untreated (constantly maintained at 28.5 °C during the course of the experiment) or heat shocked as previously described [28]. Embryos were transferred to 50-mL conical tubes containing 28.5 °C E3 medium. All but ~5 mL of the medium was removed and rapidly replaced with 45 mL of 37 °C medium. The embryos were immediately transferred to a 37 °C water bath for 1 h, with periodic gentle mixing of the solution to keep uniform bathing of the solution on the embryos. Afterwards, all but ~5 mL of the 37 °C medium was removed and quickly replaced with 45 mL of 28.5 °C medium. The embryos were allowed to recover at 28.5 °C for 1 h prior to isolating RNA.

### 2.7. RNA Isolation, Microarray Experiments, and Data Analysis

Three pools each of *n* = 50 DMSO control and propoxur-treated embryos were chilled and then homogenized on ice using the Kontes Pellet Pestle with Cordless Motor. Trizol was added (1 mL) immediately following homogenization, and total RNA was extracted according to the manufacturer’s specifications (Invitrogen, Carlsbad, CA, USA). A quality check of the total RNA was performed using an Agilent 2100 Bioanalyzer (Agilent Technologies, Palo Alto, CA, USA) and the RNA 6000 Pico Assay kit in 2011. At that time, microarray analysis was widely used due to the expense of RNA sequencing. We understand that microarray data can have increased false positives, lower detection range, and saturation of high signals compared to RNA-seq technologies. Five hundred ng of total RNA along with the TargetAMP 1-Round Aminoallyl-aRNA Amplification Kit 101 (Epicentre, Madison, WI, USA) and Agilent Spike-in Controls for one color microarrays were used to produce Aminoallyl-aRNA according to the vendor’s protocol. Five µg of each aminoallyl-aRNA sample and the Alexa fluor 555 (Molecular probes/ Life Technologies, Foster City, CA, USA) were used for the labeling step. The samples were incubated with the dye for 30 min at room temperature. After the labeling incubation, the samples were run through RNeasy Mini Elute column (Qiagen, Valencia, CA, USA) to remove all of the unincorporated dye. Then, the samples were checked on the NanoDrop and prepared for hybridization 4 × 44 K Agilent *D. rerio* oligo microarrays following Agilent “One-Color Microarray-Based GE Analysis” protocol. In the hybridization reaction 1.65 µg of Alexa 555-labeled aminoallyl-aRNA was used to hybridize on the Agilent 60-mer oligo array (Zebrafish Gene Expression V.3, 4 × 44 K) for 17 h at 65 °C at 10 rpm in a hybridization oven. A total of six microarrays were analyzed: three independent replicates for each of the DMSO and propoxur treatments. After hybridization, the slides were washed with Agilent GE Wash Buffers following Agilent’s protocol. Slides were immediately scanned with the Agilent dual laser scanner with SureScan High Resolution Technology. Tiff images were analyzed using Agilent’s feature extraction software version 10.7.1.1 to obtain fluorescent intensities for each spot on the arrays. 

Microarray data were imported into GeneSpring v12.6 (Agilent Technologies, Santa Clara, CA, USA) for analysis. The gene expression data on each microarray were quantile normalized, and replicate probes were condensed at the gene level. Filtering was performed to first select genes with expression ≥ 20th percentile in all three replicates for either DMSO or propoxur. The resultant set of genes was analyzed using RankProd [29] to identify statistically significant changes in gene expression. Using a false discovery rate cutoff of 10%, 59 genes were identified comparing DMSO and propoxur-treated samples (Supplemental Table S1). Genes of interest (defined as those with a *p* value < 0.05 and an absolute fold change > 1.3) were uploaded into Ingenuity Pathway Analysis (IPA; Qiagen Bioinformatics) software, converted to homologous human genes, and analyzed using the Agilent Probe ID as the identifier. Twenty molecules were available for pathway analysis of enriched disease and biologic functions. 

### 2.8. Real-Time Reverse Transcription (qRT)-PCR

Total RNA (1 μg) was reverse-transcribed with random hexamer primers and MuLV reverse transcriptase (Applied Biosystems). cDNA was purified using the QIAquick PCR Purification kit according to the manufacturer (Qiagen) protocols and eluted into PCR-grade deionized water. Primers specific for *hspb9* (NM_001114705; 5′- TGGACGACCCTTTCTTTGAG-3′ (forward) and 5′-GCATTATTTGGGCTCTACGG-3′ (reverse); Tm = 55 °C) and *hspb11* (NM_001099427; 5′- AGAGCTCGCCGTTAAACAG-3′ (forward) and 5′- AATAGGATCCCTTCCCATCG-3′ (reverse); Tm = 56 °C) were from [30]. Transcript levels were normalized to 18S (5′-TCGGCTACCACATCCAAGGAAGGCAGC-3′(forward) and 5′-TTGCTGGAATTACCGCGGCTGCTGGCA-3′ (reverse); Tm = 60 °C). Primers (0.5 μM final) were mixed with LightCycler 480 SYBR Green I Master Mix (Roche Applied Science) in a volume of 18 μL and added to a well of a 96-well plate. Either 2 μL of cDNA or water was then added. The samples were run on a LightCycler 480 real-time PCR machine (Roche Applied Science). Relative transcript levels were determined by the 2^−ΔΔCt^ method [31].

### 2.9. Statistical Analysis

Data were plotted using GraphPad Prism 4 software (GraphPad Software, Inc.). Three independent biological replicates were analyzed for statistical significance (*p* < 0.05) using the Student’s *t*-test for the analysis of two groups or a one-way ANOVA followed by a Bonferroni post hoc test for analysis of more than two groups. 

## 3. Results

### 3.1. Propoxur Delays Zebrafish Development in a Dose- and Time-dependent Manner

Previous studies showed that carbamate exposure had profound effects on notochord when embryos were exposed between 4–13 hpf but not between 14–24 hpf [32]. Thus, we chose to dechorionate and treat the embryos with propoxur at 15–16.5 hpf to avoid complications due to notochord distortions. First, embryos were treated with a range propoxur concentrations from 12.5–800 μg/mL at 15–16.5 hpf and then examined for viability after 24 h at 28.5 °C. No statistical differences on viability were observed between the concentrations tested (Figure 1A). We then repeated the viability analysis by comparing the percentage of viable embryos in an untreated control group, a group treated with 1% DMSO, and finally a propoxur-treated group with a concentration of 100 μg/mL. Again, no statistical differences were observed (Figure 1B). However, starting at 50 μg/mL, we noted that a significant portion of the population was developmentally delayed (Supplemental Figure S1A) and that, at 100 μg/mL, three phenotypic groups were observed: severe delay, mild delay, or no delay (Figure 1C–F; Supplemental Figure S1A). Specifically, we found that only 22.80 ± 2.26% of the embryos exposed to 100 μg/mL propoxur were at the proper developmental stage as compared with 75.33 ± 5.06% in the DMSO-treated control group (Figure 1C–F; *p* = 0.0007). Conversely, 45.53 ± 4.50% of the embryos exposed to 100 μg/mL propoxur were severely delayed compared with only 10.23 ± 3.04% of the DMSO-treated control group (Figure 1C; *p* = 0.0029). The severe delay correlated with an increase in acridine orange uptake, suggestive of increased cell death, and a decrease in the G1/S phase proliferation marker Proliferating Cell Nuclear Antigen (PCNA; Figure 1G–I), suggestive of decreased proliferation. 

Next, we tested whether the delay phenotypes were dose- and time-dependent. Specifically, we treated embryos with a range of propoxur from 12.5–800 μg/mL at 15–16.5 hpf and then examined for delay phenotypes after 24 h at 28.5 °C. At concentrations lower than 100 μg/mL, no severely delayed phenotypes were observed (Supplemental Figure S1A), whereas at concentrations greater than 100 μg/mL, a proportional trend toward severely delayed fish was detected, with nearly all of the embryos displaying the severely delayed phenotype at 800 μg/mL (Supplemental Figure S1A). In addition to a dose-dependent effect on phenotype, we observed a time-dependent effect on the phenotypic outcome of propoxur treatment. Specifically, we observed that embryos treated at 24 hpf had considerably more normal and mildly delayed animals compared with embryos treated at 15–16.5 hpf and significantly fewer severely delayed animals (Supplemental Figure S1B). In addition, embryos treated after 24 hpf showed no delay differences compared with DMSO-treated controls (data not shown). These data suggest a dose- and time-dependent developmental window of sensitivity to propoxur-induced delay. 

**Figure 2:**
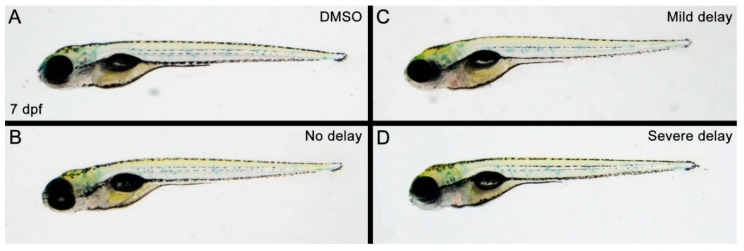
Zebrafish with propoxur-induced delays undergo physical recovery to obtain the general appearance of control zebrafish by 7 days postfertilization: (**A**) DMSO-treated control group; (**B**) propoxur-treated group exhibiting phenotype of no delay; (**C**) propoxur-treated group exhibiting phenotype of mild delay; and (**D**) propoxur-treated group exhibiting phenotype of severe delay.

After determining that a propoxur treatment concentration of 100 μg/mL and a window for treatment between 15–16.5 hpf resulted in three distinct phenotypic outcomes at 40 hpf, the next step was to examine how these fish developed. Due to the rapid embryological growth of zebrafish, 40 hpf embryos are typically still in their chorions and therefore equate to Carnegie Stage 17 (6 weeks) of human prenatal development [33,34]. Similarly, 7 dpf in zebrafish development is post-hatching and roughly corresponds to postnatal human development [35,36]. Therefore, we analyzed whether the early embryologic delay observed at 40 hpf was still present at 7 dpf in propoxur-treated zebrafish. Interestingly, we observed that the phenotypic delays that resulted from propoxur treatment at 40 hpf (Figure 1D–F) were not present at 7 dpf (Figure 2A–D). Instead, fish from the mild and severely delayed groups regained the general physical appearance of a normal 7 dpf zebrafish (Figure 2A–D). These findings were reminiscent of previous studies that found a significant correlation between prenatal exposure to the pesticides chlorpyrifos, diazinon, and propoxur (as detected by umbilical cord plasma) and reduced weight and length at birth [12] but not at later stages in development. However, since follow-up studies in humans found that prenatal propoxur exposure correlated to significantly impaired motor development by 2-years of age [15], we analyzed propoxur-treated zebrafish at 7 dpf for impaired sensory and motor defects. 

### 3.2. Zebrafish with the Severely Delayed Phenotypes Develop a Photophobic Response to Light Leading to Reduced Locomotor Activity

Behavior analysis of propoxur-treated zebrafish was performed at 7 days postfertilization (dpf) using a repeating cycle of 3-min intervals of light and dark stimuli, as it is well established that, in using this alternating paradigm, zebrafish larvae exhibit less movement during the light stimuli compared to the dark stimuli [37]. Larval fish movement was tracked during the light/dark intervals using EthoVision XT 13 software, and heat maps and statistical differences between the groups were analyzed for movement in light and dark intervals. 

**Figure 3:**
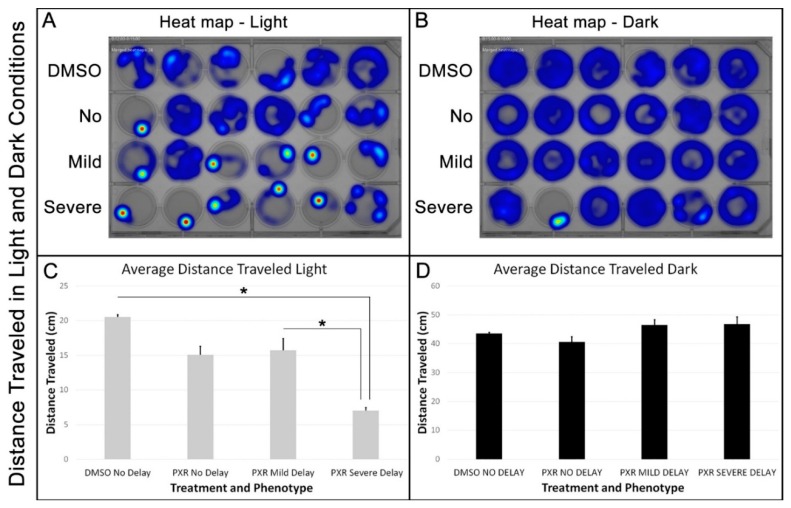
Zebrafish severely delayed by propoxur treatment showed greater aversion to light with less movement at 7 days postfertilization: (**A**) Heat map representation of the concentration of movement for each group during a single light interval; (**B**) heat map representation of the concentration of movement for each group during a single dark interval; (**C**) graphical representation of the average distance traveled for each group during the cumulative light intervals. The asterisks indicate a significant difference between the groups; and (**D**) graphical representation of the average distance traveled for each group during the cumulative light intervals.

We first analyzed the average distance moved between the groups during the light and dark intervals. Heat maps provided a visual representation of the concentration of movement of zebrafish in each of the four groups (DMSO-treated controls, no delay, mild delay, and severe delay) and confirmed that each group of larval fish moved more in the dark than in the light (Figure 3A,B). However, statistical analysis of distance traveled during each light and dark interval revealed that zebrafish in the severe delay group showed a significant photophobic response resulting in a decrease in locomotor activity compared with the no delay and mild delay groups (Figure 3C; *p* < 0.01). In contrast, no differences between the groups were observed in the average distance traveled during the dark intervals (Figure 3D; *p* = 0.36). 

**Figure 4:**
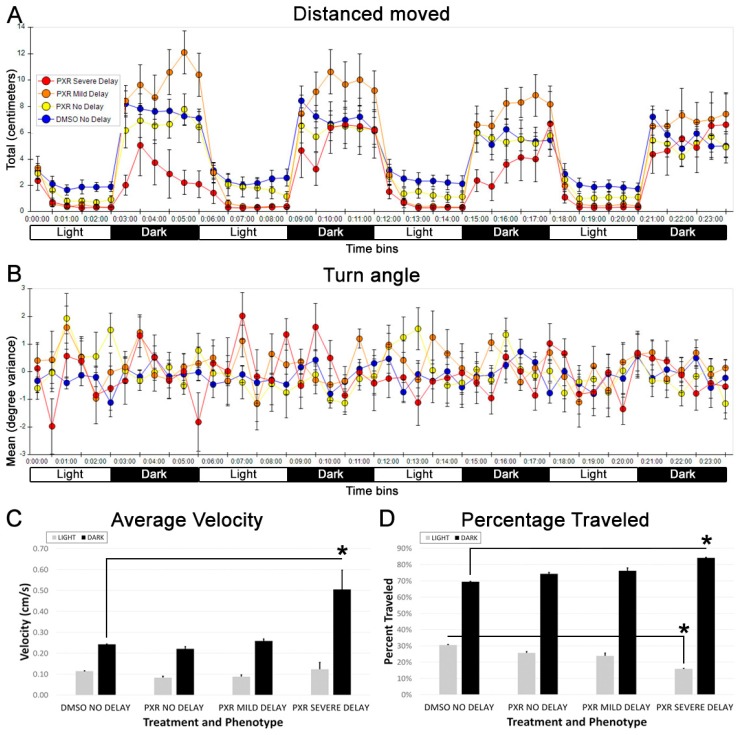
Abnormal movements observed in zebrafish at 7 dpf that were severely delayed by propoxur treatment at 40 hpf. (**A**) Graphical representation of the distance moved into 30-second bins: severely delayed fish (red circles) displayed extreme light aversion and a delayed response to movement in the dark. (**B**) Graphical representation of the turn angle into 30-second bins: severely delayed fish (red circles) displayed extreme changes in turn angle. (**C**) Graphical representation of the average velocity of movement in light and dark. (**D**) Graphical representation of the average percentage traveled in light and dark: asterisk represents statistical differences compared with group indicated in the graph (*p* < 0.01).

Next, we analyzed each group broken down into 30-second bins over the course of the light and dark intervals and analyzed differences in the percentage of time traveled in the light and the dark intervals, turn angle, and average velocity of movement. We noted that the severely delayed animals displayed an extreme photosensitive response and displayed latency in resuming movement in the dark intervals (Figure 4A). However, their overall movement recovered to within normal range of the other groups by the end of the 3 min dark interval (Figure 4A). In contrast to these clear behavioral differences, when analyzing turning behavior, no significant differences between the groups were observed (Figure 4B). Additionally, the severely delayed group showed a statistically higher average velocity of movement in the dark and an overall greater percentage of movement in the dark (Figure 4C,D), suggesting that normal movement is possible in these animals but is largely limited to movements in the dark. In support of this interpretation was the statistical analysis of “rebound” graphs, which were generated to graphically depict how the behavior of each group changed over the course of the entire 24-min alternating light/dark paradigm (Figure 3). As already noted, for all groups, the distance traveled increased with each initiation of the dark stimulus and decreased with each initiation of the light stimulus. (Figure 4A; Figure S3). However, the severely delayed zebrafish showed an increase in “rebound” dark movements with each new dark stimulus, such that the slope of the light/dark movement over the course of the experiment was significantly higher in the severely delayed group compared with the control groups (Supplemental Figure S3; *p* < 0.01). Together, these data demonstrated that zebrafish that were severely delayed by the propoxur treatment at 40 hpf displayed abnormal movements at 7 dpf despite an apparent physical recovery from the early delay. 

### 3.3. The Expression of the Small Heat Shock Proteins hspb9 and hspb11 are Increased in Propoxur Treated Embryos

**Figure 5:**
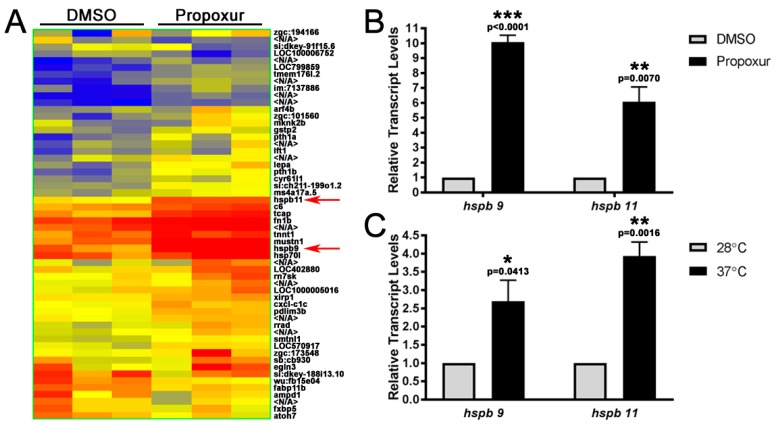
The expression of the small heat-shock proteins *hspb9* and *hspb11* are increased in propoxur-treated embryos. (**A**) Heat map of 56 genes that were identified by microarray analysis to be significantly regulated by propoxur treatment: Each column represents a separate biological pool of 40 hpf embryos treated with either DMSO or propoxur. Dark blue represents a 2-fold downregulation of expression, whereas dark red represents a 2-fold upregulation in expression. Expressions of *hspb9* and *hspb11* are indicated by red arrows. (**B**,**C**) qRT-PCR confirmation of *hspb9* and *hspb11* upregulation following propoxur treatment and heat shock: The asterisks represent different levels of significance (*p* values are shown).

As a preliminary means of elucidating gene expression changes in response to propoxur treatment, embryos were treated with DMSO or 100 μg/mL propoxur at 15–16.5 hpf and 3 separate pools of control and propoxur embryos were used for microarray analysis at 40 hpf. We identified 59 genes that were differentially regulated by propoxur with a change in expression of at least 1.3-fold or greater, with 48 genes upregulated and 11 downregulated by propoxur (Figure 5A). Ingenuity pathway analysis was performed on differentially regulated genes, as defined above (FC > 1.3, *p* value < 0.05) using Agilent Probe IDs; in the propoxur exposed embryos, 20 molecules were accessible for analysis with IPA. All differentially expressed genes are listed in Supplemental Table S1, and the top five enriched disease and biologic functions (diseases and disorders, and physiological system development and function) with specific pathways involved in cancer and organismal injury and abnormalities, neurological disease, and hematological system development and function quantity of blood cells are listed in Table 1 and Table 2. 

We chose to further examine *hspb9* and *hspb11* due to their significant upregulation by microarray analysis and their potential roles in zebrafish development [38,39]. Further examination by qRT-PCR confirmed that hspb9 and hspb11 were significantly upregulated 10.08 ± 0.456-fold (*p* < 0.0001) and 6.074 ± 0.995-fold (*p* = 0.0070), respectively, by propoxur (Figure 5B). As expected, both small heat-shock proteins were also induced by heat shock, with hspb11 being more significantly affected (3.931 ± 0.386-fold (*p* = 0.0016) versus 2.699 ± 0.573-fold (*p* = 0.0413); Figure 4C). This is in agreement with the fold expression induced by heat shock of hspb9 and hspb11 as reported elsewhere [30].

**Figure 6:**
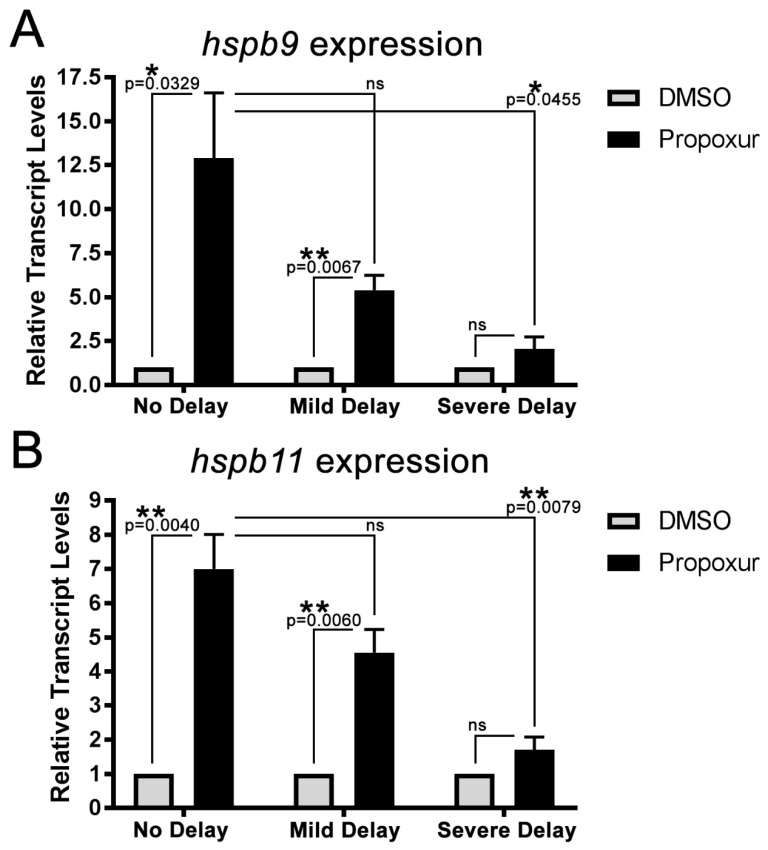
The expression of the small heat-shock proteins *hspb9* and *hspb11* correlates to the phenotypic outcome of propoxur exposure. (**A**) qRT-PCR of *hspb9* expression in DMSO- or propoxur-treated embryos based on phenotype (no delay, mild delay, and severe delay) and (**B**) qRT-PCR of *hspb11* expression in DMSO- or propoxur-treated embryos based on phenotype (no delay, mild delay, and severe delay): The asterisks represent different levels of significance (*p* values are shown), and “ns” indicates no significance between the groups. The error bars represent SEM, and *n* = 3 per group.

To begin to address if either Hspb9 or Hspb11 might contribute to establishment of the developmental delay phenotype, we manually staged and sorted propoxur-treated zebrafish embryos into the three phenotypic groups (no-delay, mild delay, and severe delay) and examined whether a correlation existed between the expression of hspb9 and hspb11 and the developmental phenotypes induced by propoxur. Compared with the DMSO treated groups, both heat-shock proteins were significantly elevated in the no delay group (hspb9: 12.90 ± 3.72 (*p* = 0.0329); hspb11: 6.998 ± 1.005-fold (*p* = 0.0067)) and the mild delay group (hspb9: 5.391 ± 0.850 (*p* = 0.0040); hspb11: 4.560 ± 0.669-fold (*p* = 0.0060)) but not in the severe delay group (hspb9: 2.10 ± 0.68 (*p* > 0.9999); hspb11: 1.70 ± 0.38-fold (*p* = 0.9208); Figure 6A,B). Transcript levels of hspb9 and hspb11 were decreased in the mild delay group relative to the no delay group in embryos treated with propoxur but were found not to be significantly different. However, transcript levels were significantly decreased in the severe delay group of propoxur-treated embryos relative to the no delay group (Figure 6A,B). Transcript levels in this severe group were not significantly different from the DMSO-treated severe group for either hspb9 (2.055 ± 0.683-fold greater than DMSO) or hspb11 (1.704 ± 0.380-fold greater than DMSO; Figure 6A,B). Together, these data show that propoxur increased the expression of these small heat-shock proteins in a given population (Figure 5B,C) but that the more severely delayed the phenotype, the less significant of an increase was observed (Figure 6A,B). This raises the possibility that decreased ability to upregulate these small heat-shock proteins in response to propoxur might cause some embryos to be more severely delayed. 

## 4. Discussion

The increasing global usage of pesticides has allowed many chemicals of concern to persist in the environment. Pesticide exposures have been linked to several negative human health effects, such as asthma; cancer; and disruption of the nervous, endocrine, and reproductive systems [40,41,42,43,44,45,46]. Young children and pregnant women are particularly sensitive to pesticide exposures [5,7,47,48,49,50,51], which have been linked to delays in human fetal development and lifelong neurotoxic disabilities [52,53,54,55,56]. Recognizing the risks posed by pesticide exposure highlights the need for further investigation into the underlying molecular mechanisms of action and evidence-based programs of prevention [57]. 

Zebrafish have become a highly used model to study pesticide toxicity, and previous reports have demonstrated that insecticide exposure causes developmental delays in zebrafish [19,32,58,59,60]. We decided to focus our analysis on the role of propoxur in development given its worldwide use and the shift towards carbamate insecticides [1]. Here, we showed that exposure of zebrafish embryos to propoxur during a defined window of development resulted in three distinct developmental delay phenotypes. These delayed phenotypes are labeled as no delay, mild delay, and severe delay and reflect a chemically induced disturbance in embryogenesis. In general, developmental delays are an observed phenomenon usually consisting of a reversible phenotypic response and nonspecific effects [61,62,63,64]. Despite these delays being phenotypically reversible, persistent exposures to teratogenic substances allows them to act for a longer time, intensifying the severity of harm and yielding deficits in function that are difficult for an organism to overcome [65,66]. This observation is seen in animal and human studies, but our knowledge of the etiological pathways and all of the biological processes impacted is limited [67,68,69,70,71,72,73,74]. 

In order to understand the complex developmental deficits caused by propoxur exposure, we employed a two-factor approach by examining zebrafish genetics and behavior. First, we used a microarray analysis, which identified 59 genes that were differentially regulated. The IPA results from this microarray highlight genes involved in cancer, inflammatory response, neurological, and cardiovascular disease. It is worth noting that half of the genes in this data set are related to cancer. For some time, there has been an increasing incidence of cancer in children aged 0–14 years globally [75,76,77]. Leukemia is one of the most prevalent pediatric cancers, and despite medical advancements, it continues to be a global burden [77,78]. The increasing global occurrence of leukemia is also tied to a large body of literature implicating increasing pesticide use as an addressable and preventable etiology [45,78,79,80,81,82,83,84,85,86]. In our past study, prenatal exposure to propoxur was associated with detection of the leukemia-associated translocation t(8;21) in cord blood cells [87]. Aside from establishing programs to reduce exposure risk to pesticides, it is also worth identifying genetic differences that render certain children more susceptible. This is where future studies using the zebrafish model can be used to help understand the roles of genes involved and their exact contributions to leukemia progression. 

Amongst these genes of interest, *hspb9* and *hspb11* were significantly upregulated. Heat-shock proteins play an important role as molecular chaperones in protein folding during normal development and in response to external stressors. Small heat-shock proteins (sHSPs) are found in both humans and fish, with ten sHSPs identified in humans and an additional three sHSPs identified in zebrafish [30]. Most of the thirteen sHSPs are expressed during zebrafish development, including hspb9 and hspb11 [30,88]. In our analysis, we found that expression of *hspb9* and *hspb11* correlated with the phenotypic outcomes to propoxur exposure. The no-delay and mild-delay groups had elevated heat shock protein expression, but the severely delayed zebrafish did not. This suggests that most of the embryos are able to respond to the toxicant stressor with increased sHSP expression, allowing development to proceed normally. However, if embryos are unable to rally an upregulation of sHSPs during development, a developmental delay phenotype is observed. Klüver et al. reported similar results in hspb11 expression with propoxur and also demonstrated that hspb11 expression is muscle activity dependent, which is modulated by AChE inhibitors [89]. A lack of hspb11 upregulation in response to an AChE inhibitor in embryos significantly interferes with neuromuscular transmission between motor neurons and muscle fibers [90] and could begin to explain some of the behavioral alterations witnessed in our study. 

Investigating changes in behavior, especially locomotor activity, has become a widely accepted method for assessing compounds of interest for toxicological and neurodevelopmental issues [91,92,93,94,95,96,97,98]. Zebrafish are an excellent species for modeling neurobehaviors associated with human disease because the neurotransmitter systems are well described and there is conserved function with respect to the human nervous system [99,100,101,102]. Our behavioral assay, also known as the light–dark locomotion test, uses an automated tracking platform to record zebrafish behavior in alternating cycles of light and dark stimuli [103]. Zebrafish larvae usually have higher locomotor activity in the dark cycle compared to the light, which is in contrast with adult zebrafish where locomotor activity in response to light is reversed. Our results show a photosensitive response in the severely delayed phenotype, which is a unique behavioral pattern that has not been reported, to the best of our knowledge, in larval zebrafish. This photosensitive phenotype is reminiscent of the clinical manifestation of photosensitive epilepsy in humans [104]. Under normal conditions with respect to brain function, nervous system development, and visual pathways, the behavioral pattern in response to the light/dark stimuli resembles a symmetrical wave pattern. However, after exposure to sublethal concentrations of environmental toxicants, this behavioral pattern is disturbed. In our results, locomotor activity remains constant in the light cycles; however, once propoxur is introduced, behavior in the dark becomes more and more dysregulated. The severely delayed propoxur fish are significantly different from the controls and display increasingly dysregulated behavior over time. This increase in behavior over time could be a reflexive seizure response induced by the white-light stimulus. Using a zebrafish behavioral protocol outlined by Baraban et al. future studies could examine propoxur-induced behavior, focusing on behavioral patterns and locomotor activity to investigate if specific seizure stages were reached [105]. The behavioral findings of the current study also coincide with the results of our IPA analysis which identified 3 human genes of interest: SOCS3, TSPO, and UBE3A related to seizures. In humans, the neonatal phase is the most vulnerable period of life for developing seizures [106] and could easily be provoked by pesticide exposure. Similar to fetal alcohol spectrum disorder (FASD), which produces a variety of neurological disorders (i.e., epilepsy in prenatally exposed children [107]), insecticides like propoxur could also contribute to formation of neurological disorders and behavioral alterations. Further studies will be required to assess the relationship between AChE inhibitors like propoxur and exposure during development that could provide a new etiology for reflex seizures/epilepsy.

The mechanism of action for the major commercial insecticides involves three nervous system targets: AChE (organophosphates and carbamates), voltage-gated sodium channels (pyrethroids), and the acetylcholine receptor (neonicotinoids) [108,109,110]. Although propoxur’s mechanism of action is inhibition of AChE, this cannot explain all of its effects on development, indicating that there are off-target effects not previously considered [111]. This also demonstrates a gap in chemical testing for neurodevelopmental toxicity, something we intend to address using the zebrafish animal model. To address these disparities in chemical testing, it would be advantageous to begin studying the different behavioral phenotypes caused by pesticide exposure in zebrafish. Larval zebrafish have a smaller behavioral repertoire compared to adults, but there is a significant number of behaviors that can be exploited for neuroscience gains [103,112,113,114]. Taking this into consideration, larval and adult zebrafish behavioral studies should be used in parallel compared to stand alone approaches [113]. Furthering this research, knock out and transgenic lines using CRISPR should be used to elucidate genetic factors and gene-environment interactions of pesticides in development. This research approach will provide an opportunity to gather evidence that early exposure to pesticides like propoxur is linked to abnormal behavior and neurological dysfunction in life. 

## Figures and Tables

**Table 1:** Top five diseases and biological functions generated by Ingenuity Pathway Analysis (IPA) of genes with significant changes in gene expression in control versus propoxur-exposed groups with the number of genes and percentage of genes in the dataset.

IPA Top Diseases and Biological Functions	
Diseases and Disorders	Number of Molecules/% of Genes in the Dataset
Organismal Injury and Abnormalities	14/70%
Cancer	10/50%
Neurological Disease	7/35%
Cardiovascular Disease	6/30%
Inflammatory Response	5/25%
**Physiological System Development and Function**	
Hematological System Development and Function	7/35%
Cardiovascular System Development and Function	4/20%
Organismal Development	10/50%
Visual System Development and Function	3/15%
Skeletal and Muscular System Development and Function	5/25%

**Table 2:** Diseases and functions from IPA of genes with the most significant changes (*p* value) in gene expression in control versus propoxur-exposed groups, including the genes in the pathways.

Diseases and Functions Annotation	*p* Value	Genes
**Cancer and Organismal Injury and Abnormalities**		
Cancer of secretory structure	3.41 × 10^−2^	*C6, CSRP3, DARS, FBP2, HSD3B7, IQCH, LGALS1, SOCS3, TSPO, UBE3A*
Advanced malignant tumor	4.68 × 10^−3^	*CSRP3, LGALS1, SOCS3, TSPO, UBE3A*
Metastasis	1.55 ×10^−2^	*CSRP3, LGALS1, SOCS3, UBE3A*
**Neurological Disease**		
Seizures	8.55 × 10^−3^	*SOCS3, TSPO, UBE3A*
Amyotrophic lateral sclerosis	2.05 × 10^−2^	*FBP2, TSPO*
Damage of nervous system	2.45 × 10^−2^	*SOCS3, TSPO*
Neurodegeneration	4.10 × 10^−2^	*C6, UBE3A*
**Hematological System Development and Function**		
Quantity of blood cells	6.75 × 10^−4^	*C6, LGALS1, NPR3, SNAI3, SOCS3, TSPO*
Quantity of leukocytes	2.77 × 10^−3^	*C6, LGALS1, SNAI3, SOCS3, TSPO*
Quantity of phagocytes	8.89 × 10^−4^	*C6, LGALS1, SNAI3, SOCS3*
Quantity of lymphocytes	6.93 × 10^−3^	*LGALS1, SNAI3, SOCS3, TSPO*

## Supplementary Materials

The following are available online at https://www.mdpi.com/2305-6304/7/4/50/s1, Figure S1: Propoxur-induced delay is dose and time-dependent, Figure S2: Apparatus and software used for zebrafish behavioral analysis, Figure S3: Zebrafish severely delayed by propoxur-treatment continued to show aversion to light but exhibited increasing movement in dark stimuli over time at 7 days postfertilization, Table S1: All 59 differentially regulated genes by propoxur.
