# Supplementary material for: Exposure of Larval Zebrafish to the Insecticide Propoxur Induced Developmental Delays that Correlate with Behavioral Abnormalities and Altered Expression of hspb9 and hspb11
Source: Toxics. 2019 Sep 21;7(4):50. doi: 10.3390/toxics7040050 (PMC6958418; doi:10.3390/toxics7040050)
Supplement: Supplementary file 1 [file toxics-07-00050-s001.pdf]

Supplementary Materials: Exposure of Larval Zebrafish to the Insecticide Propoxur Induced Developmental Delays that Correlate with Behavioral Abnormalities and Altered Expression of *hspb9* and *hspb11*

Jeremiah N. Shields, Eric C. Hales, Lillian E. Ranspach, Xixia Luo, Steven Orr, Donna Runft, Alan Dombkowski, Melody N. Neely, Larry H. Matherly, Jeffrey Taub, Tracie R. Baker and Ryan Thummel

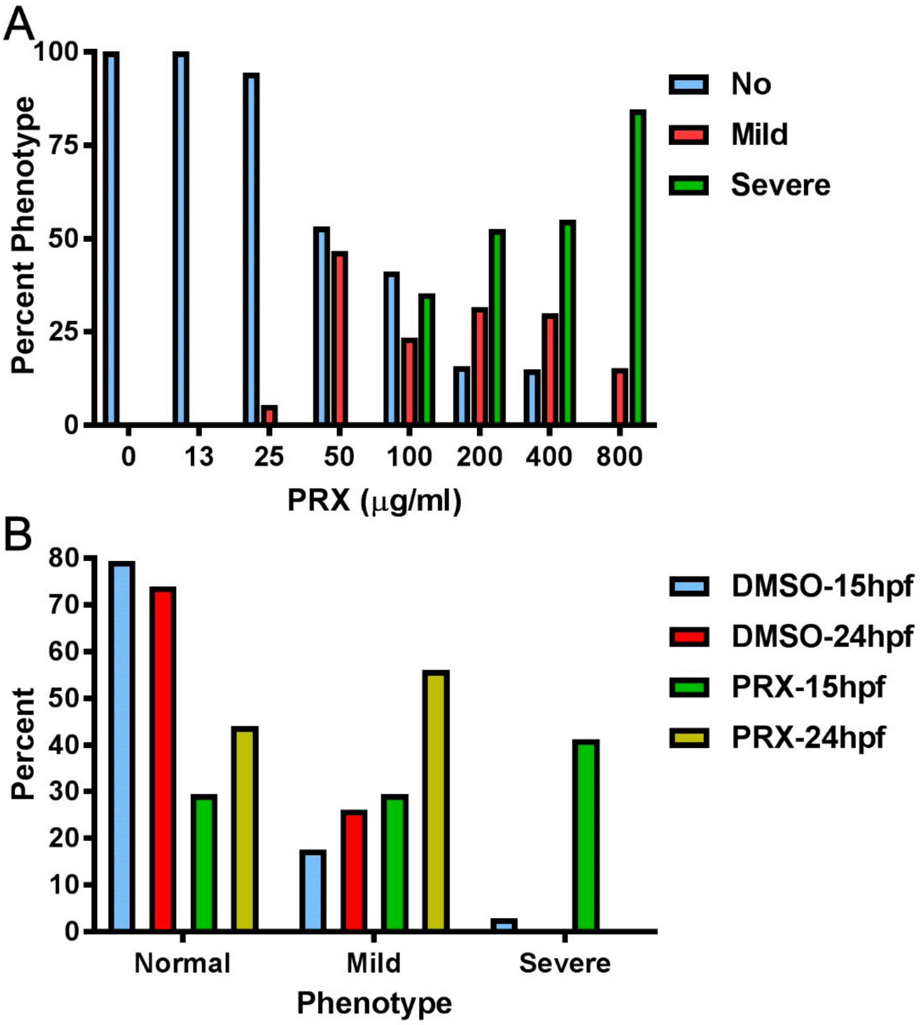

Figure S1. Propoxur-induced delay is dose and time-dependent. (**A**) Differing concentrations of propoxur elicited varying distributions of phenotypes. (**B**) Propoxur elicited varying distributions of phenotypes depending on time of treatment in hours postfertilization (hpf).

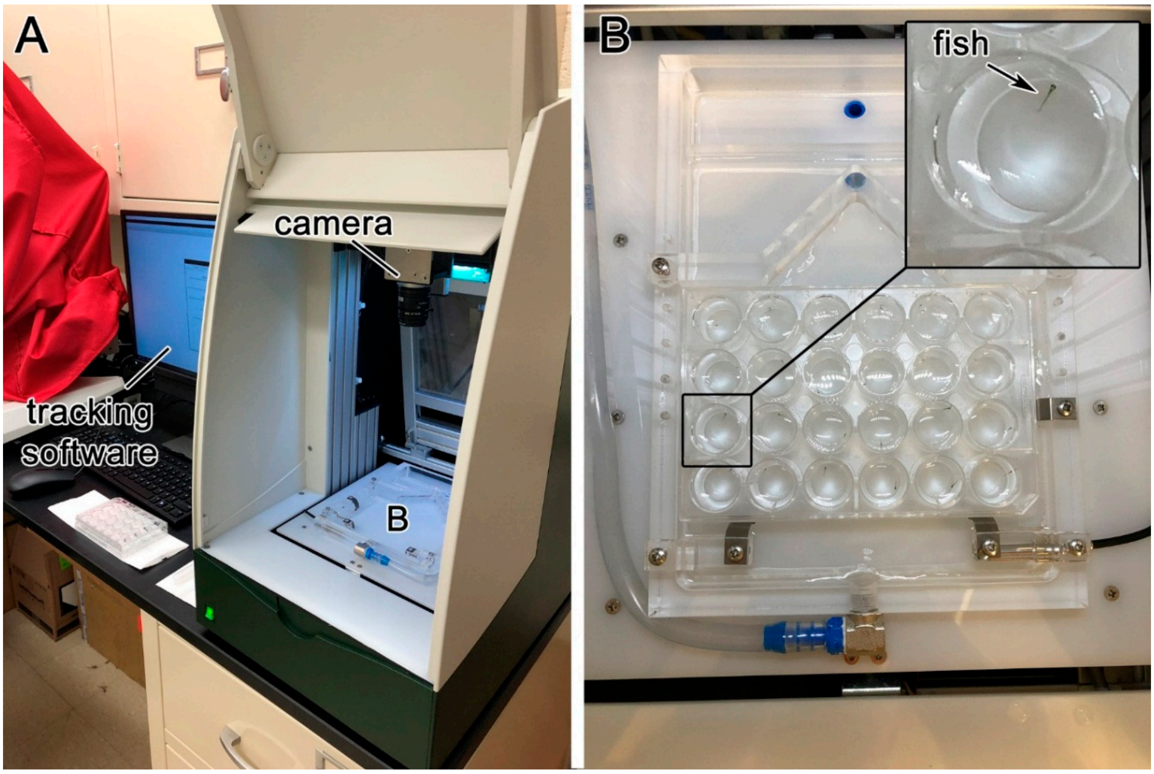

Figure S2. Apparatus and software used for zebrafish behavioral analysis. (**A**) Noldus DanioVision Observation Chamber including Basler Gen1 tracking camera with connected EthoVision XT 13 software and (**B**) individual fish placed in a single well of a 24-well plate and subjected to a steady stream of flowing water to maintain constant temperature during experimentation.

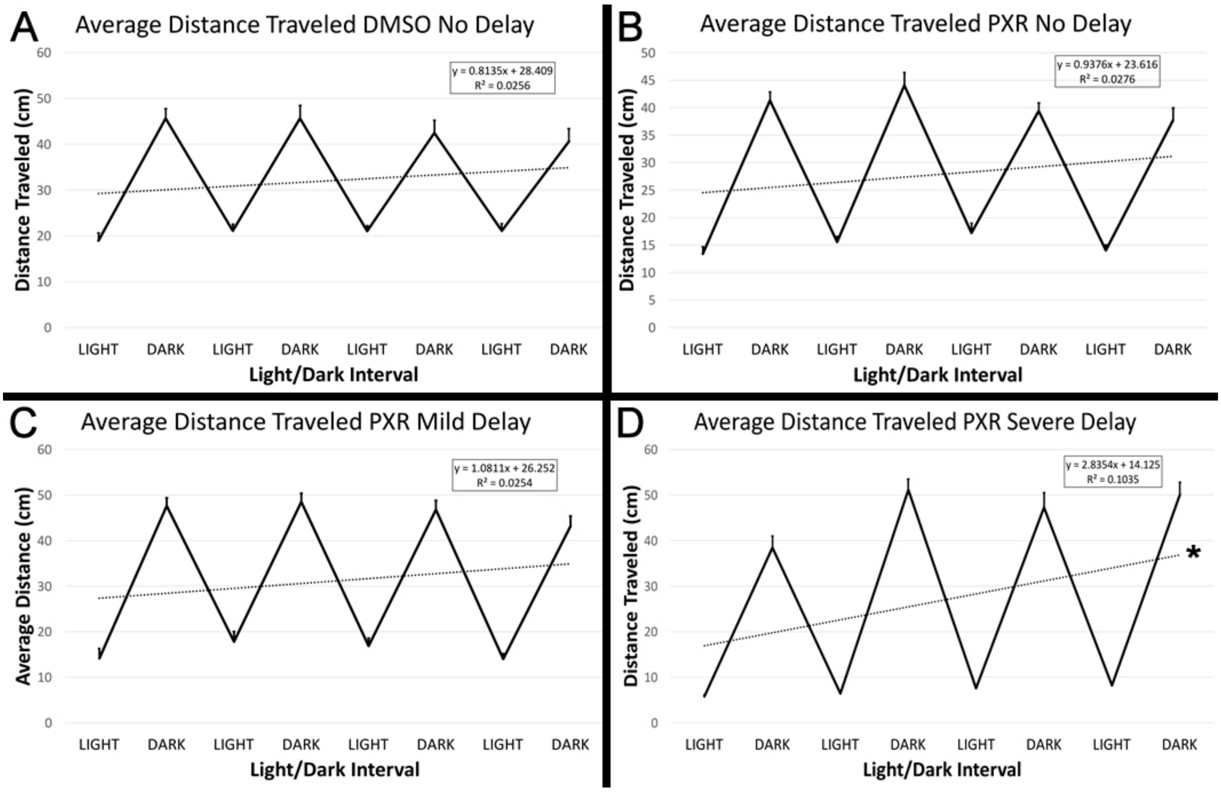

Figure S3. Zebrafish severely delayed by propoxur-treatment continued to show aversion to light but exhibited increasing movement in dark stimuli over time at 7 days postfertilization. (**A**) movement in each light/dark interval for the DMSO-treated control group; (**B**) movement in each light/dark interval for the propoxur-treated no-delay group; (**C**) movement in each light/dark interval for the propoxur-treated mild-delay group; and (**D**) movement in each light/dark interval for the propoxur-treated severe-delay group. The asterisk indicates a significant difference compared with the DMSO control group ($p < 0.01$).

Table S1. All 59 differentially regulated genes by propoxur.

Probe Name	Gene Symbol	Gene Name	Fold Change	*p* Value (Treatment)
**A_15_P167696**	*lepa*	leptin a	7.902	7.64×10^{-5}
**A_15_P196091**	*hspb11*	heat shock protein, alpha-crystallin-related, b11	7.597	1.27×10^{-7}
**A_15_P131406**	*hspb9*	heat shock protein, alpha-crystallin-related, 9	7.654	4.89×10^{-5}
**A_15_P622046**	*pth1b*	parathyroid hormone 1b	6.880	9.99×10^{-5}
**A_15_P658521**	*pth1b*	parathyroid hormone 1b	6.707	7.30×10^{-5}
**A_15_P115624**	*pth1b*	parathyroid hormone 1b	6.705	9.36×10^{-5}
**A_15_P665381**	*mustn1*	musculoskeletal, embryonic nuclear protein 1	3.669	2.87×10^{-5}
**A_15_P177461**	*mustn1*	musculoskeletal, embryonic nuclear protein 1	3.817	3.44×10^{-5}
**A_15_P696841**	*mustn1*	musculoskeletal, embryonic nuclear protein 1	3.782	3.97×10^{-5}
**A_15_P101018**	*pdlim3a*	PDZ and LIM domain 3a	3.497	1.41×10^{-5}
**A_15_P353060**	*LOC799859*	dystrobrevin binding protein 1-like	3.727	6.73×10^{-5}
**A_15_P344195**	*c6*	complement component 6	3.419	4.08×10^{-5}
**A_15_P648726**	*fn1b*	fibronectin 1b	2.692	1.11×10^{-4}
**A_15_P153516**	*pdlim3b*	PDZ and LIM domain 3b	3.069	6.63×10^{-8}
**A_15_P628085**	*c6*	complement component 6	2.900	6.40×10^{-5}
**A_15_P186611**	*LOC570917*	actin-binding Rho activating protein-like	2.863	5.79×10^{-5}
**A_15_P267296**			3.534	5.15×10^{-5}
**A_15_P183421**	*LOC567047*	apolipoprotein B-editing catalytic subunit 2b	2.241	2.78×10^{-5}
**A_15_P147861**	*zgc:113342*	zgc:113342	2.787	1.54×10^{-5}
**A_15_P489412**			2.445	2.67×10^{-5}
**A_15_P484040**	*socs3b*	suppressor of cytokine signaling 3b	2.128	5.39×10^{-5}
**A_15_P170166**	*xirp1*	xin actin-binding repeat containing 1	2.403	3.99×10^{-6}
**A_15_P109841**	*zgc:123218*	zgc:123218	2.131	4.04×10^{-5}
**A_15_P448890**	*xirp1*	xin actin-binding repeat containing 1	2.398	2.97×10^{-5}
**A_15_P665301**	*zgc:103438*	zgc:103438	1.794	4.88×10^{-5}
**A_15_P186201**	*LOC100331199*	troponin T2, cardiac-like	2.025	7.48×10^{-5}
**A_15_P144831**	*LOC557301*	cocaine and amphetamine regulated transcript protein type I-like	2.064	2.64×10^{-6}
**A_15_P529407**	*snai3*	snail homolog 3	1.996	1.00×10^{-4}
**A_15_P119140**	*csrp3*	cysteine and glycine-rich protein 3 (cardiac LIM protein)	1.868	7.75×10^{-6}
**A_15_P183376**			1.682	5.76×10^{-5}
**A_15_P183361**	*LOC100333780*	F-box protein 6-like	1.740	5.85×10^{-5}
**A_15_P153201**	*iqch*	IQ motif containing H	2.123	7.80×10^{-5}
**A_15_P721587**	*ms4a17a.7*	membrane-spanning 4-domains, subfamily A, member 17A.7	1.564	3.25×10^{-5}
**A_15_P398890**			1.933	9.68×10^{-5}

**A_15_P652341**	*ms4a17a.7*	membrane-spanning 4-domains, subfamily A, member 17A.7	1.572	3.67×10^{-5}
**A_15_P540642**	*wu:fb12e11*	wu:fb12e11	1.654	9.43×10^{-6}
**A_15_P620376**	*ms4a17a.7*	membrane-spanning 4-domains, subfamily A, member 17A.7	1.538	3.86×10^{-5}
**A_15_P308956**	*LOC567640*	similar to laminin alpha 3	1.609	6.28×10^{-5}
**A_15_P663057**	*lgals2a*	lectin, galactoside-binding, soluble, 2a	1.482	9.96×10^{-5}
**A_15_P670046**	*wu:fb12e11*	wu:fb12e11	1.540	7.97×10^{-6}
**A_15_P462225**	*LOC100330405*	Membrane-spanning 4-domains subfamily A member 8A-like	1.591	6.07×10^{-7}
**A_15_P182651**	*si:dkey-184p18.2*	si:dkey-184p18.2	1.672	5.89×10^{-6}
**A_15_P310476**	*il13ra1*	interleukin 13 receptor, alpha 1	1.592	7.36×10^{-5}
**A_15_P750606**	*LOC100330405*	Membrane-spanning 4-domains subfamily A member 8A-like	1.512	1.14E-05
**A_15_P147091**	*npr3*	natriuretic peptide receptor C/guanylate cyclase C (atrionatriuretic peptide receptor C)	1.411	4.68×10^{-5}
**A_15_P233056**	*hsd3b7*	hydroxy-delta-5-steroid dehydrogenase, 3 beta- and steroid delta-isomerase	1.419	3.36×10^{-5}
**A_15_P736276**	*dars*	aspartyl-tRNA synthetase	1.520	1.21×10^{-4}
**A_15_P296716**	*tspo*	translocator protein	1.381	1.09×10^{-4}
**A_15_P190176**	*ubiad1*	UbiA prenyltransferase domain containing 1	−1.136	1.01×10^{-5}
**A_15_P655546**	*ube3a*	ubiquitin protein ligase E3A	−1.324	8.15×10^{-5}
**A_15_P195142**	*zgc:165604*	zgc:165604	−1.586	1.17×10^{-4}
**A_15_P154381**	*zgc:136906*	zgc:136906	−1.533	1.42×10^{-5}
**A_15_P149841**	*epyc*	epiphycan	−1.708	7.88×10^{-5}
**A_15_P624136**	*epyc*	epiphycan	−1.749	1.24×10^{-4}
**A_15_P149081**	*fbp2*	fructose-1,6-bisphosphatase 2	−2.110	1.31×10^{-4}
**A_15_P629426**	*zgc:136906*	zgc:136906	−2.144	1.06×10^{-4}
**A_15_P557497**	*LOC100003805*	CD98 solute carrier family 3 member 2-like	−2.173	6.47×10^{-5}
**A_15_P186936**	*LOC566281*	tripartite motif protein 21-like	−3.859	1.07×10^{-4}
**A_15_P107927**	11	11	−5.130	8.74×10^{-5}
